# Pathologische Ruptur des distalen Peronäalretinaculums mit Luxation der M.-peronaeus-longus-Sehne bei einem Hochsprungolympioniken: Eine 15-Jahres-Kasuistik

**DOI:** 10.1007/s00113-025-01566-x

**Published:** 2025-04-02

**Authors:** Hans Zwipp

**Affiliations:** UniversitätsCentrum für Orthopädie, Unfall- und Plastische Chirurgie, Universitätsklinikum Carl Gustav Carus, Technische Universität Dresden, Fetscherstraße 74, 01307 Dresden, Deutschland

**Keywords:** Lokale Kortison-Injektion, Retinaculum mm. peroneorum inferius, Retinaculum-Ruptur, Processus trochlearis-Hypertrophie, Periostlappenplastik, Distal retinaculum of peroneus tendon, Pathological rupture, Peroneal tubercle hypertrophy, Peroneus longus tendon dislocation, Periosteal flap

## Abstract

**Hintergrund:**

Hypertrophie der Trochlea peronaealis und/oder eine lokale Kortisoninjektion begünstigten eine pathologische Ruptur des unteren distalen peronäalen Retinaculums mit Luxation der M.-peronaeus-longus-Sehne.

**Ziel der Arbeit:**

Vorstellung einer distalen Peronäalretinaculumersatzplastik mittels Periostlappen nach Remodellierung der hypertrophierten Trochlea peronaealis zur dauerhaften Führung der M.-peronaeus-longus-Sehne.

**Material und Methode:**

Ein 21-jähriger Hochsprungathlet erlitt beim Rechtskurvenlauf ohne jedes Trauma ein schmerzhaftes Knacken am linken äußeren Fuß. Klinisch bestand ein schmerzhaftes, palpatorisch reproduzierbares Überspringen einer Peronäalsehne bei übergroßer Trochlea peronaealis. Fünf Monate zuvor war wegen Schmerzen eine lokale Kortisoninjektion erfolgt. Röntgen, CT und MRT zeigten eine erhebliche Hypertrophie der Trochlea peronaealis links mit reitender Luxation der M.-peronaeus-longus-Sehne auf ihr. Bei bekannter insuffizienter konservativer Therapie bestand die Indikation zur Operation.

**Ergebnis:**

Im 15-Jahres-Verlauf bestand kein Rezidiv. Im Folgejahr der Operation wurde der Patient deutscher Meister im Hochsprung und weitere 5 Jahre später deutscher Meister im Dreisprung.

**Diskussion:**

Ob allein die Hypertrophie der Trochlea peronaealis und/oder die lokale Kortisoninjektion die pathologische Ruptur des distalen Retinaculums mit Luxation der Sehne bedingten/bedingte, bleibt offen. Eine sichere Sehnenführung mit Wiederherstellung der vollen Sprungkraft ist nur operativ zu erzielen.

## Abstract (en)

**Background:**

Hypertrophy of the peroneal tubercle and/or a local cortisone injection promoted a pathological rupture of the distal peroneal retinaculum with dislocation of the peroneus longus tendon.

**Objective:**

Presentation of a distal peroneal retinaculum replacement using a periosteal flap after remodelling of the hypertrophied peroneal tubercle by creating the natural sulcus below it, which enable safe and permanent guidance of the peroneus longus tendon.

**Material and method:**

A 21-year-old high jump athlete suffered a painful crack on the outer left foot while running a right hand curve without any trauma. Clinically, there was a painful, palpable reproducible dislocation of the peroneus longus tendon probably due to an extremely oversized peroneal tubercle. A local cortisone injection had been given 5 months previously because of local pain. The X‑ray, computed tomography (CT) and magnetic resonance imaging (MRI) examinations showed substantial hypertrophy of the left peroneal tubercle with a riding dislocation of the peroneus longus tendon on it. As conservative therapy was known to be insufficient, surgery was indicated.

**Result:**

There was no recurrence over the course of 15 years. In the year following the operation, the patient became German champion in the high jump discipline and another 5 years later, German champion in the triple jump.

**Discussion:**

If hypertrophy of the peroneal tubercle and/or the local cortisone injection alone caused the pathological rupture of the distal retinaculum with dislocation of the tendon remained unclear. A secure and longstanding tendon guidance with restoration of full jumping strength can only be surgically achieved.

## Einleitung

Während Luxationen der Peronäalsehnen bei Ruptur des proximalen Retinaculums der Peronäalsehnen am Außenknöchel akut oder chronisch, insbesondere bei dessen flacher peronäaler Rinne im Breiten- und im Hochleistungssport häufig zu behandeln und zahlreiche operative Methoden hierzu bekannt sind, ist die Ruptur des unteren Teils des distalen Retinaculums der Peronäalsehnen mit Luxation der M.-peronaeus-longus-Sehne eine sehr seltene Entität, die bisher nur fallweise beschrieben wurde [4, 8, 11, 12, 18]. Rupturen der M.-peronaeus-longus-Sehne sind ebenso extrem selten und treten am ehesten dann auf, wenn nahe zur Umschlagstelle der Sehne zum Os cuboideum ein Os peronaeum als Sesambein in der Sehne interponiert ist oder eine lokale Kortisoninjektion vorausging [5, 6, 17].

## Anatomie, Biomechanik und Pathophysiologie

*Anatomisch-biomechanisch* bildet die M.-peronaeus-longus-Sehne gemeinsam mit der Sehne des M. tibialis anterior den wichtigen Steigbügel des Fußes [19]. Der M. peronaeus longus ist mit 1,7 mKg ein noch stärkerer Pronator des Fußes als sein benachbarter M. peronaeus brevis mit 1,3 mKg Arbeitsleistung, wobei beide mit 0,4 bzw. 0,3 mKg auch Plantarflektoren sind [10]. Während die Sehne des M. peronaeus brevis ausschließlich lateral verläuft und an der Tuberositas ossis metatarsale V ansetzt, verläuft die Sehne des M. peronaeus longus bis zum Os cuboideum etwa zwei Drittel ihrer Wegstrecke ebenfalls lateral, um aber dort unter das Os cuboideum zu tauchen und in dessen plantarer Furche durch den Canalis plantae [19] bzw. den Sulcus tendinis m. peronei longi [10] zum medialen Fußrand umzuschwenken und schließlich plantar am Cuneiforme I sowie an den Metatarsalia I und II zu inserieren. Dadurch senkt der M. peronaeus longus den medialen Fußrand, während der M. peronaeus brevis den lateralen Fußrand hebt [10, 19], konzertiert in pronatorischer Wirkung. Nach Root et al. [16] bewirkt der M. peronaeus longus zusätzlich bei Inversion des Rückfußes durch die Superimposition des Os cuneiforme laterale gegenüber dem Os cuboideum eine signifikante Anhebung und Stabilisierung des Lisfranc-Quergewölbes. Bei Kontraktion des M. peronaeus longus führt seine Sehne unter dem Hypomochlion von Cuboid und Cuneiformia einen stark plantarflektierenden Zug auf den ersten Fußstrahl aus. Deshalb ist es sprachlich korrekter, von einer vorgeschalteten Trochlea peronaealis (lat. trochlea: Flaschenzug, Winde) im Sinne einer Umlenkrolle, als von einem Tuberculum peronaeale (lat. tuberculum: Höckerchen) zu sprechen. Dies gilt v. a. für das „peroneal tubercle“, das in der angloamerikanischen Literatur verwendet wird. Anderson [1] weist hingegen auf die primäre und sekundäre Bedeutung hin. Für die M.-peronaeus-brevis-Sehne allein betrachtet, wäre „Tuberculum“ ausreichend, da diese in den Windenmechanismus nicht einbezogen ist. Dem Autor ist zudem zur Anatomie des *Retinaculum peronaeale distale* wichtig darauf hinzuweisen, dass dieses einen oberen Teil zur Führung der M.-peronaeus-brevis-Sehne und einen unteren Teil zur Führung der M.-peronaeus-longus-Sehne hat, da bei Luxation der Longussehne nur der untere Teil gerissen, der obere intakt beschrieben wird [4, 8, 11, 12, 18]. Im vorliegenden Fall einer pathologischen Luxation bei extremer Hypertrophie der Trochlea peronaealis und vorausgegangener Kortisoninjektion war der obere Teil des distalen Retinaculums ebenfalls unbeschädigt; der untere Teil ließ keinerlei Reste einer retinakulären Struktur erkennen.

*Pathophysiologisch* ist zu beachten, dass bei allen Sprungdisziplinen nach beschleunigendem Anlauf, Absprung, Flugphase und Landung immer ein *einbeiniger Absprung* erfolgen muss [15]. Somit ist dieser Sprungfuß stets extremen mechanischen Belastungen ausgesetzt, wodurch die anatomisch relativ kleine Trochlea peronaealis resp. das Tuberculum peronaeale [1] durch den sehr häufigen Absprungstress im Training überbeansprucht wird, wodurch der repetitive hohe Anpressdruck der Sehne gegen das Areal der Trochlea peronaealis zu erheblicher Reibung mit konsekutiv-schmerzhafter Periostitis und/oder Peritendinitis führen kann, was auch bei Langstreckenläufern beobachtbar ist [13, 14]. Ähnliche Belastungen dürften ebenso im Hochleistungssport wie Fußball und Skaten pathogenetisch gegeben sein [4, 8, 11, 12, 18].

## Fallvorstellung

*Anamnestisch*^1^ nahm der Patient bereits als 17-jähriger Athlet an nationalen und internationalen Wettkämpfen im Hochsprung teil, erreichte mit 21 Jahren bei den Olympischen Spielen in Peking mit 2,32 m den 5. Platz und erzielte bei den Weltmeisterschaften in Berlin mit wiederholter Sprunghöhe eine Bronzemedaille. Dieses Ergebnis gelang ihm, wenngleich er zu diesem Zeitpunkt bereits Schmerzen bei Anlauf und Absprung am linken äußeren Fußrand hatte. Diese Schmerzregion wurde u. a. mit einer einmaligen lokalen Kortisoninjektion behandelt. Fünf Monate später, im Dezember 2009, verspürte der Athlet während eines Leichtathletiktrainings in einer Rechtskurve ohne Anlass ein schmerzhaftes Knacksen an seinem linken Fuß, weshalb er sich vorstellte.

*Klinisch* konnte am linken Fuß eine sehr vergrößerte Trochlea peronaealis getastet werden. An deren Oberrand war offensichtlich eine Sehne nach fußrückenwärts luxiert; diese konnte unter passiver Supination des Fußes fußsohlenwärts reponiert werden. Bei aktiver Pronation des Fußes reluxierte die Sehne schmerzhaft.

**Abb. 1:**
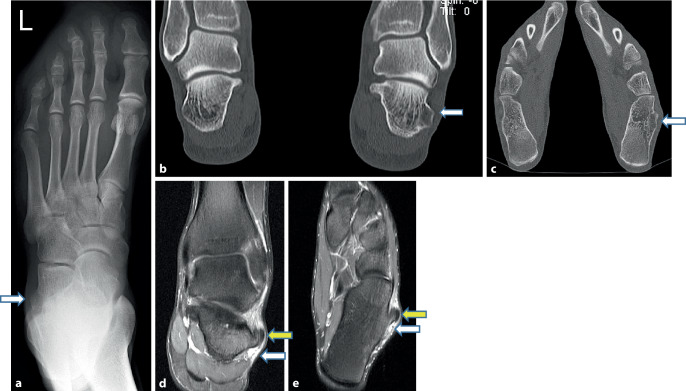
Röntgen‑, CT- und MRT-Befunde, **a** Das dorsoplantare Röntgenbild zeigt die hypertrophierte Trochlea peronaealis links (*Pfeil*), **b**, **c** koronarer und axialer CT-Schnitt zeigen im Seitenvergleich die erhebliche ossäre Hypertrophie der Trochlea peronaealis links mit 6–8 mm Differenz (*Pfeile*), **d**, **e** koronarer und axialer MRT-Schnitt zeigen die Luxation der M.-peronaeus-longus-Sehne nach kranial auf Trochlea und M.-peronaeus-brevis-Sehne reitend (*gelber Pfeil*) sowie das leere Fach unterhalb der Trochlea peronaealis (*weißer Pfeil*)

*Bildgebung* (Abb. 1a–e) zeigte sich im dorsoplantaren *Röntgen*bild (Abb. 1a) die erhebliche ossäre Hypertrophie der Trochlea peronaealis (*weißer Pfeil*) links. Das präoperative *CT *ließ im Seitenvergleich in den koronaren Schnitten (Abb. 1b), mehr noch als auf den axialen (Abb. 1c), die enorme exostotische Protuberanz der Trochlea auf doppelte Größe (*weißer Pfeil*) gegenüber der gesunden rechten Seite erkennen. Das zur Weichteilbeurteilung zusätzlich erstellte *MRT* zeigte im koronaren Schnitt (Abb. 1d) die leere Tasche unterhalb der Trochlea mit fehlendem Retinaculum, einen nur dünnen Synovialschlauch (*weißer Pfeil*) sowie die Superimposition der luxierten M.-peronaeus-longus-Sehne auf die M.-peronaeus-brevis-Sehne mit deren erhaltenem Retinaculum (*gelber Pfeil*). Eine axiale Schicht (Abb. 1e) belegte analog die Superimposition (*gelber Pfeil*) der luxierten M.-peronaeus-longus-Sehne auf die darunter liegende, normal in ihrem Retinaculum geführte M.-peronaeus-brevis-Sehne.

## Therapie

**Abb. 2:**
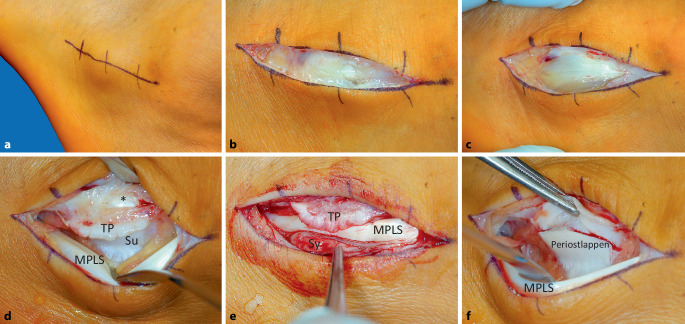
Intraoperativer Befund: **a** eingezeichnete, 3–4 cm lange Hautinzision direkt über der prominenten Trochlea peronaealis, **b** die Synovia über der luxierten Sehne ist erbsgroß aufgerissen, **c** nach Spaltung der Synovia wird die auf der Trochlea peronaealis und der kranial davon lokalisierten in situ liegenden M.-peronaeus-brevis-Sehne als breit aufgespannte, reitend luxierte M.-peronaeus-longus-Sehne erkennbar, **d** die mit einem Spatel nach kaudal weggehaltene M.-peronaeus-longus-Sehne (*MPLS*) lässt jetzt die blumenkohlartig ausgezogene Trochlea peronaealis (*TP*) an deren oberflächlichster Ebene erkennen sowie den unmittelbar darunter abgeflachten Sulcus (*Su*). Die M.-peronaeus-brevis-Sehne (*Asteriskus*) in situ schimmert durch ein dünnes oberes Retinaculum hindurch, **e** nach Reposition der *MPLS* unter den Kragen der *TP* wird die oberflächliche Lage der *MPLS* erkennbar. Reste eines Retinaculums fehlen komplett, und die untere Hälfte der Synovia (*Sy*) kann substanziell keine Führung der Sehne übernehmen, **f** deshalb wird jetzt das Periost direkt unterhalb der Umschlagstelle inzidiert, um die Stärke des hebenden Periostlappens zu prüfen

Bei gegebener Indikation zur Operation fand sich 5 Tage nach pathologischer Ruptur **intraoperativ** bei direktem Zugehen über der ausladenden Trochlea peronaealis mittels leicht nach plantar geneigter Inzision der Haut über 3–4 cm Länge (Abb. 2a) prima vista ein nur erbsgroß aufgerissener Synovialschlauch mit darunter liegender luxierter M.-peronaeus-longus-Sehne (Abb. 2b), die nach Längsspaltung der synovialen Hülle deutlich hervortrat (Abb. 2c). Durch das Zugehen durch den Synovialschlauch trat der dicht fußsohlenwärts, parallel zur Sehne verlaufende N. cutaneus dorsalis lateralis, Ast des N. suralis, zu keinem Zeitpunkt der Operation in Erscheinung. Durch instrumentelles Weghalten der luxierten Sehne fußsohlenwärts wurde die leere, zu gering ausgeprägte Rinne unterhalb der blumenkohlartig aufgetriebenen Trochlea peronaealis erkennbar (Abb. 2d). Die M.-peronaeus-longus-Sehne ließ sich leicht reponieren, zeigte aber bei quasi fehlender Rinne keine sichere Führung unter dem verschmälerten Dach der Trochlea. Außer einem dünnen Synovialschlauch (Abb. 2e) waren keine Residuen eines distalen Retinaculums vorhanden, weshalb es bei diesem pathologischen Befund notwendig erschien, eine vertiefte Rinne für den Lauf der M.-peronaeus-longus-Sehne und einen stabilen Retinaculumersatz mittels distal breit gestieltem Periostlappen zur Wiederherstellung der Anatomie und Biomechanik zu schaffen.

## Remodellierung der Trochlea peronaealis, Sehnenreposition und Periostlappenersatz für den führenden unteren Anteil des Retinaculum peronaeale distale

**Abb. 3:**
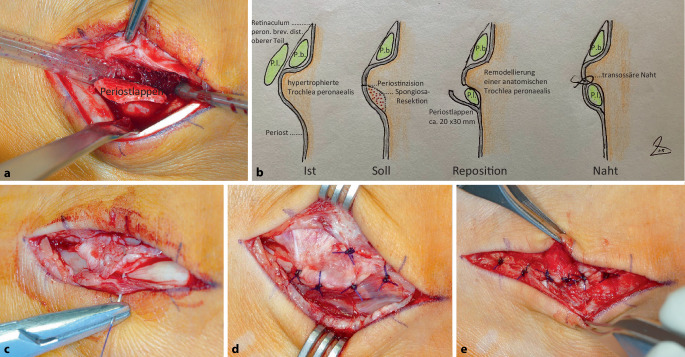
Operatives Vorgehen: **a** Das kräftige Periost (2 mm) wird kranial horizontal auf 20 mm Breite und seitlich jeweils auf 30 mm Länge vertikal nach sohlenwärts bis auf den Knochen inzidiert und anschließend mit dem Raspatorium vom Calcaneus abgeschoben, **b** die Operationsskizze zeigt den *Ist-Zustand* mit intaktem oberen Teil des Retinaculum tendinis m. peronei brevis und korrekt positionierter Sehne des M. peronaeus brevis (P.b) sowie mit luxierter, auf der hypertrophierten Trochlea peronealis reitenden Sehne des M. peronaeus longus (P.l.), den *Soll-Zustand* mit notwendiger Remodellierung eines Sulcus unter Resektion von überschüssiger Spongiosa mit scharfem kleinen Meißel und Löffel sowie gekrümmter feiner Knochenfeile. *Reposition* der Sehne in den Neosulcus und *Naht* des Lappens transossär an die Trochlea sind schematisch, seitlich gesehen, gezeichnet (**c**, **d**), intraoperativer Situs der transossären Naht des Periostlappens mit abschließender Synovialnaht (**e**)

Zur sicheren Führung der M.-peronaeus-longus-Sehne wird im ersten Schritt zum Ersatz des unteren Retinaculumanteils direkt an der Umschlagskante der Trochlea ein 20 mm breiter Perioststreifen inzidiert (Abb. 2f). Dieser wird mit feinem Meißel und Raspatorium vom Kalkaneus nach fußsohlenwärts um ca. 3 cm abgeschoben (Abb. 3a, b). Im zweiten operativen Schritt wird jetzt unter Weghalten des kräftigen Periostlappens eine vertiefte Rinne für einen glatten Lauf der M.-peronaeus-longus-Sehne mittels feinem Meißel, scharfem Löffel und feiner Knochenfeile geschaffen, wodurch die hypertrophierte Fläche unter der Trochlea peronaealis, jetzt um mindestens 5 mm vertieft, einer anatomisch-physiologischen Trochlea peronaealis gleicht (Abb. 3b, Ist-Soll-Reposition-Naht). Nach Reposition der luxierten Sehne in ihr neues Bett wird im dritten Schritt mit transossärer Naht der Periostlappen an die Trochlea peronaealis als Ersatz für den unteren Teil des distalen Peronäalretinaculums (Abb. 3b–d) befestigt. Nach feiner Naht des Synovialschlauchs (Abb. 3e) wird die Haut verschlossen.

**Postoperativ** erfolgte die Nachbehandlung funktionell mit Mobilisation und Protektion des linken Fußes in einer Caligamed-Knöchelschiene (Bauerfeind AG, Zeulenroda) für 5 Wochen unter zunehmender Vollbelastung. Frühe Übungen außerhalb der Schiene sollten nur aktive Plantarflexion und Dorsalextension umfassen, Fußkreiseln im Sinne der Pronation und Supination erst ab der 6. Woche postoperativ.

## Ergebnis

**Abb. 4:**
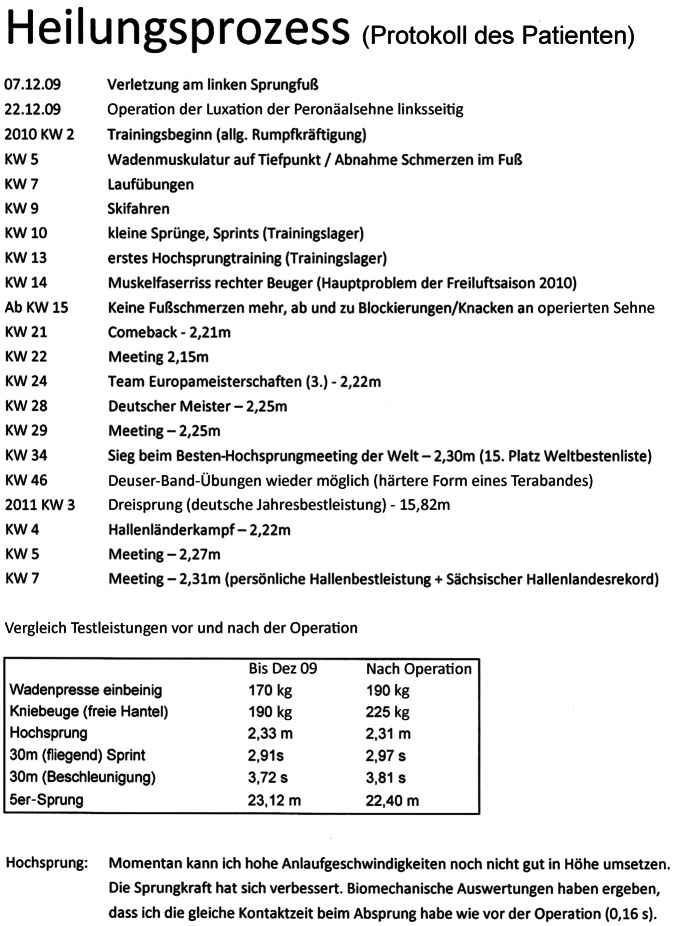
Verlaufsprotokoll vom Patienten selbst 14 Monate postoperativ erstellt und von ihm im Rahmen des 15-Jahres-Interview ausdrücklich zur vorliegenden Veröffentlichung schriftlich genehmigt. Äußerst erstaunlich sind die unten im Kasten gelisteten Testleistungen vor und nach der Operation

Nach blandem Heilverlauf konnte der Patient entsprechend seinem selbst erstelltem Protokoll zum Heilverlauf seines linken Sprungfußes (Abb. 4) nach schrittweisem Trainingsaufbau bereits nach 4 Monaten im Trainingslager ein erstes Hochsprungtraining absolvieren. Bereits 31 Wochen postoperativ (KW 28 in Abb. 4) wurde der Patient mit 2,25 m im Hochsprung Deutscher Meister. Beachtlich sind die in diesem Protokoll unten gelisteten 6 Testleistungen vor und nach der Operation, wovon 4 Parameter postoperativ sogar besser waren als präoperativ.

*15 Jahre postoperativ* gab der Patienten im brieflichen Interview an, 2012 mit 2,32 m Sieger der Deutschen Hallenmeisterschaften und 2015 Deutscher Meister im Dreisprung geworden zu sein. Er habe 2016 seine Profikarriere beendet, sei zwischenzeitlich verheiratet, habe Sohn und Tochter und arbeite als Manager in der Energiewirtschaft. Außerdem habe er 2020 als „Nichtprofisportler“ bei den Berlin-Brandenburgischen Meisterschaften nicht nur die Goldmedaille im Hochsprung, sondern auch die Bronzemedaille im Stabhochsprung gewonnen.

Bezüglich seines linken Fußes habe er postoperativ im gesamten Verlauf keinerlei Schmerzen oder Beschwerden gehabt. Er habe 2021 etliche Hochsprungwettkämpfe mit maximal 2,25 m bestritten und nähme seit 2023 gezielt an Wettkämpfen in den Wurfdisziplinen (Kugel, Diskus) teil.

## Diskussion

*Epidemiologisch* ist die isolierte Luxation der M.-peronaeus-longus-Sehne auf Höhe des distalen Retinaculums eine seltene Entität, meist als Kasuistik beschrieben. Tendinopathien der M.-peronaeus-longus-Sehne sind in 89 % der Fälle bei Patienten mit Cavovarus-Deformität des Fußes beobachtbar [3]. Zur Frage der Luxation der M.-peronaeus-longus-Sehne auf Höhe des distalen Retinaculums bei Patienten mit Tendinopathie bei Pseudohypertrophie des Tuberculum peronaeale konnte Lohrer 2019 in einem systematischen Review lediglich in 3 von 14 Artikeln nur insgesamt 5 von 25 Patienten registrieren, die eine distale M.-peronaeus-longus-Luxation in Kombination mit einer Läsion des distalen Retinaculums hatten [11], wobei diese keine Hypertrophie des Tuberculum peronaeale hatten.

*Pathomorphologisch *wurden von Hyer et al. 2005 [7] an 114 Fersenbeinen Größe und Form des Tuberculum peronaeale ermittelt und eine Klassifikation erstellt. In nur 90,4 % war überhaupt eine Trochlea peronaealis vorhanden. Davon war am häufigsten eine flache Form in 42,7 %, eine prominente in 29,1 %, eine konkave in 27,2 % und eine tunnelförmige Form in nur 1 % der Fälle nachweisbar. Auslösende Pathologien der M.-peronaeus-longus-Sehne ordneten die Autoren am ehesten den konkaven, tunnelförmigen und je nach Ausmaß den hypertrophen Formen zu.

*Sportartenspezifisch* scheint es keine Präferenz für diese Entität zu geben. So ereignete sich die 2011 erstmals beschriebene Luxation der M.-peronaeus-Sehne bei adäquatem Trauma beim Fußballsport [8]. Sie wurde ca. 8 Wochen nach erfolgloser konservativer Behandlung mit einer Retinaculumplastik mittels dorsolateral gestieltem Periostlappen versorgt. Das Tuberculum peronaeale wurde von den Autoren nach Hyer [7], nicht als hypertroph, sondern als *konkav* klassifiziert. Die volle Sportfähigkeit bestand nach 6 Monaten.

2012 beschrieben El Rassi et al. [4] eine isolierte Luxation der M.-peronaeus-longus-Sehne auf das „calcaneal tubercle“ bei einer 23-jährigen Landesmeisterin im Ice-Skating. Die Autoren fanden nach 18 Monaten Beschwerdeverlauf keine hypertrophierte Trochlea peronaealis, keine Sehnenläsion, aber ein vollständiges Fehlen des unteren Anteils des distalen Peronäalretinaculums. Anamnestisch war keine lokale Kortisoninjektion vorausgegangen. Den Defekt ersetzten die Autoren nach Reposition der Sehne durch die vordere Hälfte des oberen Anteils des distalen Retinaculums, wodurch die volle Leistung der Athletin im Ice-Skating wieder möglich wurde.

2013 berichteten Staresinic et al. [18] in einer Beobachtungszeit von 12 Jahren (2001–2011) über 3 eigene Fälle professioneller Fußballspieler, die eine M.-peronaeus-longus-Luxation nach isolierter Ruptur des distalen Retinaculums erlitten hatten. Diese wurden mit Resektion des Tuberkels (wenngleich nicht hypertrophiert), einer Rinnenbildung für jede der beiden Peronäalsehnen und zusätzlicher Retinaculumplastik erfolgreich versorgt.

Lohrer beschrieb 2020, ein Jahr nach seinem Review [11], einen eigenen Fall [12] eines 25-jährigen, internationalen Top-Level-Skaters mit partieller Luxation der M.-peronaeus-longus-Sehne, Spaltung der Sehne und knöchernem Ausriss des distalen Retinaculums. Nach Reposition und Glättung der Sehne sowie Vertiefung der Rinne wurde die Avulsion des Retinaculums transossär erfolgreich refixiert.

*Pathogenetisch* scheinen für die Entstehung einer Ruptur des distalen Retinaculums der Peronäalsehnen mit Luxation der M.-peronaeus-longus-Sehne in den bisher beschriebenen 7 Fällen extreme sportliche Belastungen des Fußes wie beim professionellen Fußball [8, 18], Skaten [4, 12] oder in den Sprungdisziplinen verantwortlich zu sein. So wirken z. B. beim Hochsprung eine Vertikalkraft bis zum 8Fachen und eine Horizontalkraft bis zum 3Fachen des Körpergewichts bei extrem kurzer Absprungzeit von 100–200 ms auf den Fuß ein [2]. Abgesehen von der Sportart weicht der hier vorgestellte 7. Fall einer isolierten Luxation der M.-peronaeus-longus-Sehne bei pathologischer Ruptur des unteren Anteils des distalen Peronäalretinaculums von den übrigen wegen der erheblich hypertrophierten Trochlea peronaealis und der vorausgegangenen Kortisoninjektion ab. Während im einzig weiblichen Fall der Ice-Skaterin [4] mit völligem Fehlen des unteren Anteils des distalen Retinaculums eine pathomorphologische Parallele zum eigenen Fall bestand, ossäre Hypertrophie und Kortisonapplikation jedoch fehlten, erscheinen die Fragen zur Genese einer pathologischen Luxation der M.-peronaeus-longus-Sehne eher mehr als weniger zu werden.

*Pathomechanisch* gilt zu bedenken, dass die M.-peronaeus-longus-Sehne durch ihre fast rechtwinklige Umlenkung von proximal-lateral nach distal-medial zuvor die Trochlea peronaealis wie ein Hypomochlion passiert, in dem sie durch die Fasern des unteren Anteils des distalen Retinaculums nach außen hin und durch die kalkaneare Wand und überdachende Trochlea nach innen und oben wie gefesselt geführt wird. Durch hohe repetitive Zug‑/Druckmomente bei jedem Abstoß des Fußes sind Mikrorisse des überlasteten Knochenareals im Sinne der Ermüdung möglich, die örtlich eine reaktive Knochenhypertrophie und/oder eine schmerzhafte Periostitis oder zusätzliche Tendinitis führen könnte, wie auch bei Langstreckenläufern beobachtet [13, 14], auslösen können.

*Pharmakologisch* greift bei diesen Pathologien eine lokale Kortisonapplikation nicht ursächlich an, sondern führt zu einer „Mesenchymnarkose“ [9]. Durch den schwindenden Schmerz werden Training und Wettbewerb fortgeführt, sodass es zur pathologischen Luxation oder Ruptur der Sehne kommen kann [6]. In Analogie wurden pathologische Rupturen der Achillessehne nach paratendinöser Kortisoninjektion beobachtet, weshalb vor dieser Maßnahme gewarnt wurde [5, 9, 20].

*Operationstechnisch* ist allen Fällen die erfolgreiche Wiederherstellung der vollen Sportfähigkeit gemeinsam, wenngleich unterschiedliche, der jeweiligen Pathologie angepasste Operationstechniken verwendet wurden.

## Fazit für die Praxis

Die vorliegende Kasuistik zeigt zum einen, dass eine Kortisonapplikation nicht nur in Sehnen-, sondern auch in Retinaculumnähe gewebeschädigend sein kann, weshalb vor diesem Therapieversuch zu warnen ist. Bei fehlendem Retinaculum peronaeorum inferius ist ein 20 mm distal breit gestielter, ortsansässiger Periostlappen als autologes Gewebe ein idealer Ersatz zur sicheren und zur dauerhaften Führung der M.-peronaeus-longus-Sehne. Besteht zusätzlich zum Verlust des Retinaculums eine ossäre Hypertrophie der Trochlea peronaealis, sollte in Analogie zum Außenknöchel bei proximaler Peronäalsehnenluxation eine zusätzliche ausreichend tiefe knöcherne Rinne geschaffen werden, um so selbst bei höchster funktioneller Beanspruchung im Hochleistungssport ein Rezidiv sicher zu vermeiden.

## Footnotes

1 Der Patient stimmte ausdrücklich allen Nennungen von Wettkämpfen, Jahreszahlen und Ehrungen schriftlich zu
